# Meta-analysis and systematic review of the relationship between sex and the risk or incidence of poststroke aphasia and its types

**DOI:** 10.1186/s12877-024-04765-0

**Published:** 2024-03-04

**Authors:** Ting-ting Li, Ping-ping Zhang, Ming-chen Zhang, Hui Zhang, Hong-ying Wang, Ying Yuan, Shan-lin Wu, Xiao-wen Wang, Zhong-guang Sun

**Affiliations:** 1 School of Rehabilitation Medicine, Shandong Second Medical University, Weifang, China; 2 Shanghai University of Medicine & Health Sciences, Shanghai University of Traditional Chinese Medicine, Shanghai, China

**Keywords:** Sex, stroke, Poststroke aphasia, aphasia type, incidence rate

## Abstract

**Objective:**

To analyse and discuss the association of gender differences with the risk and incidence of poststroke aphasia (PSA) and its types, and to provide evidence-based guidance for the prevention and treatment of poststroke aphasia in clinical practice.

**Data sources:**

Embase, PubMed, Cochrane Library and Web of Science were searched from January 1, 2002, to December 1, 2023.

**Study selection:**

Including the total number of strokes, aphasia, the number of different sexes or the number of PSA corresponding to different sex.

**Data extraction:**

Studies with missing data, aphasia caused by nonstroke and noncompliance with the requirements of literature types were excluded.

**Data synthesis:**

36 papers were included, from 19 countries. The analysis of 168,259 patients with stroke and 31,058 patients with PSA showed that the risk of PSA was 1.23 times higher in female than in male (OR = 1.23, 95% CI = 1.19–1.29, *P* < 0.001), with a prevalence of PSA of 31% in men and 36% in women, and an overall prevalence of 34% (*P* < 0.001). Analysis of the risk of the different types of aphasia in 1,048 patients with PSA showed a high risk in females for global, broca and Wenicke aphasia, and a high risk in males for anomic, conductive and transcortical aphasia, which was not statistically significant by meta-analysis. The incidence of global aphasia (males vs. females, 29% vs. 32%) and broca aphasia (17% vs 19%) were higher in females, and anomic aphasia (19% vs 14%) was higher in males, which was statistically significant (*P* < 0.05).

**Conclusions:**

There are gender differences in the incidence and types of PSA. The risk of PSA in female is higher than that in male.

**Supplementary Information:**

The online version contains supplementary material available at 10.1186/s12877-024-04765-0.

## Introduction

Stroke is the second leading cause of death in the world, and poststroke aphasia (PSA) is a common sequela of stroke patients [1, 2]. PSA is a speech disorder caused by the impairment of the language function area of the dominant hemisphere. The incidence of stroke is increasing year by year, from 13.34% in 2003 to 21.94% in 2014 and 29.55% in 2021 [3, 4]. The factors influencing PSA deserve exploring as an important factor predicting recovery or death after stroke [5, 6].

The severity and age of stroke can predict the risk of PSA [7, 8], and the location and size of stroke have an important reference role in predicting PSA types [9]. But whether gender plays a predictive role in the incidence of  PSA and its types is still controversial [10, 11]. The lateralization of cerebral hemisphere use and the onset age of stroke affect the incidence of PSA in different sexes [12]. At present, it is considered that global aphasia and broca aphasia are the most common types of PSA. But the incidence and risk of PSA between different sexes are inevitably biased the accuracy of the conclusions drawn only by comparing between groups [13]. The research on PSA focuses on curative effect, and there are few studies on the incidence and related influencing factors. In this paper, through the meta-analysis of binary variables and rates, the differences of risk and incidence between different sexes in PSA and its types are discussed in order to provide evidence-based guidance for the prevention and early rehabilitation of PSA.

## Methods

This meta-analysis was registered on the PROSPERO platform with the registration number CRD42022369411: https://www.crd.york.ac.uk/prospero/display_record.php?RecordID=369411

### Data sources and eligibility criteria

The Embase, PubMed, Cochrane Library and Web of Science were searched from January 1, 2000, to December 1, 2023. The keywords "Stroke", "Cerebral Hemorrhage", "Brain Infarction" and "Aphasia" were combined with their free words in the database. We searched for publications without restrictions on language, or type.

### Inclusion criteria

The inclusion criteria were as follows: (1) The research type was observational research. (2) The total number of stroke and aphasia cases and the number of people of different sexes were included. (3) The number of people corresponding to different sexes with PSA was included.

Exclusion criteria: (1) The data of the total number of stroke or aphasia or the number of gender is missing. (2) Inclusion or exclusion of a specific type of aphasia. (3) Aphasia caused by brain trauma, tumor, inflammation, neurodegeneration and other non-stroke causes, and specific relevant data cannot be extracted. (4) Publishing with duplicate data. (5) The data is contradictory, and the original data cannot be obtained after contacting the author. (6) The literature types are non-observational studies such as conferences, scientific and technological achievements, case reports and reviews.

### Study selection and data collection processes

According to the inclusion and exclusion criteria, the two authors independently screened the literature and were finally included in the study after browsing the topic primary selection and reading the full text. Four authors collected basic research materials and data, including the basic characteristics of the literature and the number of stroke and aphasia cases in different sexes. In case of dispute, another author re-evaluated it and reached an agreement through discussion.

### Quality evaluation

Newcastle–Ottawa Scale was used to evaluate the quality of the included cohort studies and case–control studies, including the selection of study population, comparability between groups and measurement of exposure factors/results. The total score is 9 points, 3 points and below are low quality, 4–6 points are medium quality, and 7 points and above are high quality. The quality evaluation of cross-sectional study uses the evaluation standards of American health care quality and research institutions, with a total of 11 items, in which "Yes" is 1 point, "Unclear" and "No" are 0 points, 3 points and below are low quality, 4–7 points are medium quality, and 8 points and above are high quality. The quality evaluation of all included studies was independently completed by two authors. If there are different opinions, the final result would be decided after full discussion.

### Statistical analysis

Stata 16.0 was used for analysis, meta-analysis of binary variables was used to evaluate the risk of PSA and its types, and meta-analysis of rate was used to calculate the incidence of PSA and its types. The results of continuous variables are expressed by the mean ± standard deviation, the binary variables by the odds ratio (OR), and the confidence interval (CI) by 95%. The heterogeneity (I^2^ ≤ 50%) was analysed by a fixed effect model, and when I^2^ > 50%, it was considered that the heterogeneity was large, and the random effect model was selected to analyse the data. The difference was statistically significant when *P* ≤ 0.05.

## Results

### Description of studies

**Fig. 1:**
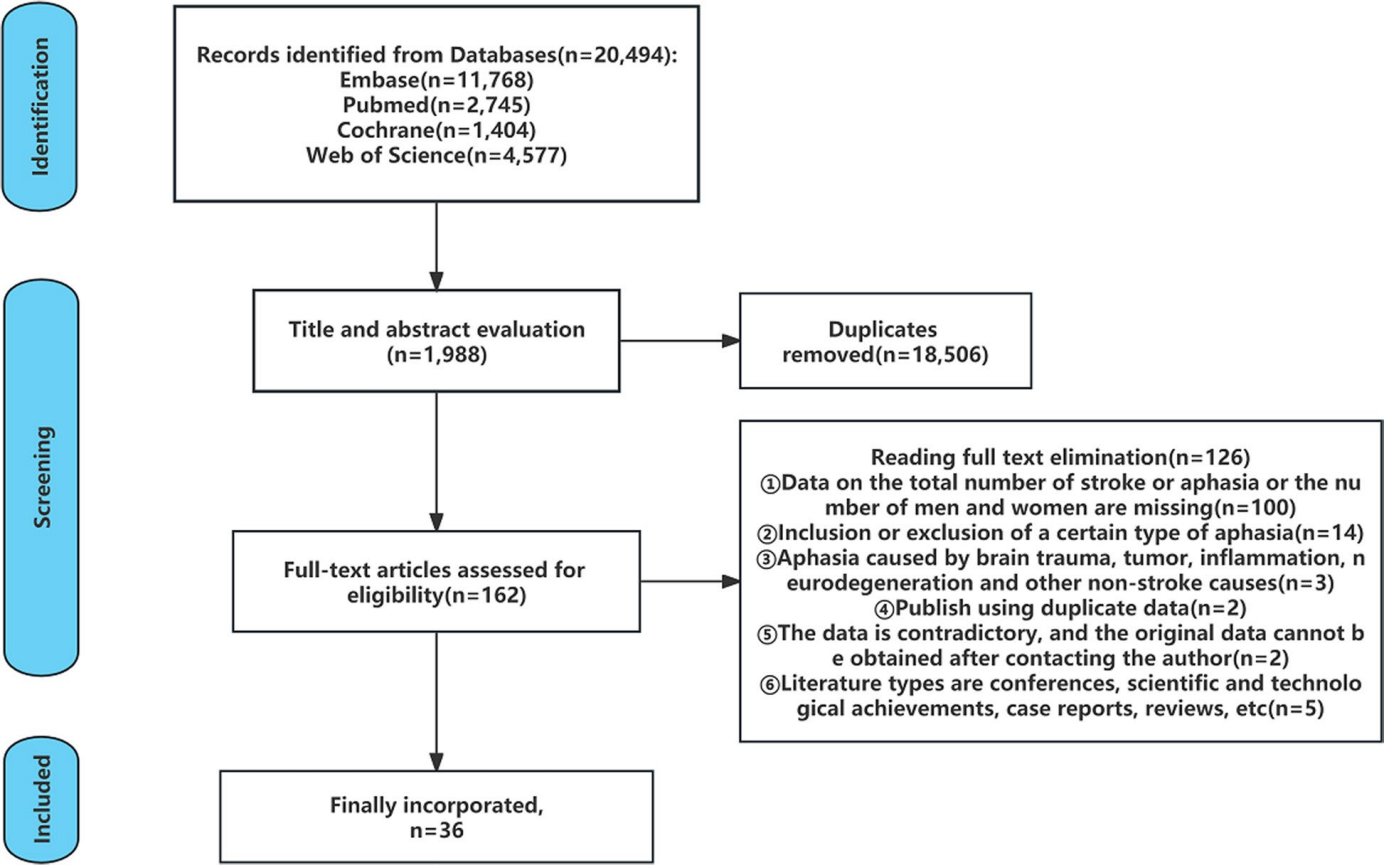
Flow diagram

After eliminating duplicate and irrelevant searches, 1988 documents were selected by reading topics and abstracts, and the remaining 36 documents met the requirements by intensive reading of 162 documents. Refer to Fig. 1 for the screening process. Among them, there were 168,259 cases of cerebral stroke (97,081 males and 71,175 females) and 31,058 cases of PSA (17,432 males and 13,626 females) in 31 studies.

There were 1048 patients (544 males and 504 females) in 7 articles involving different sexes and PSA types. There were 300 cases of global aphasia (male 134, female 166), 184 cases of broca aphasia (male 95, female 89), 196 cases of anomic aphasia (male 112, female 84), 142 cases of wernicke aphasia (male 74, female 68), 28 cases of transcortical motor aphasia (male 16, female 12), 48 cases of conductive aphasia (male 28, female 20), 36 cases of transcortical mixed aphasia (male 22, female 14) and 43 cases of transcortical sensory aphasia (male 26, female 17). There were 67 cases of other aphasia, including 9 cases of crossed aphasia (male 2, female 7), 4 cases of isolated aphasia (male 1, female 3), 3 cases of basal ganglia aphasia (male 1, female 2) and the classification of 51 cases of aphasia (male 29, female 22) was unclear.

The people included in the study came from Canada, Italy, Greece, Japan, Croatia, Norway, Britain, the United States, Sweden, Switzerland, Austria, Brazil, Belgium, Denmark, India, Chile, Spain, France and China. In 19 countries, the number of patients with left cerebral ischemic stroke is the largest, and the incidence of stroke is diagnosed by tomography or magnetic resonance imaging. The PSA was judged by the Boston Diagnostic Aphasia Test, Western Aphasia Test, Differential Diagnosis of Aphasia in Minnesota, National Institutes of Health Stroke Scale (NIHSS), Aachen aphasia test, Canadian Neurometric Scale, French Aphasia Screening Test, Pet-name ruby Afasi Norwegian Standard, AIIMS aphasia examination test, and Quick Aphasia Battery. See Table 1.

**Table 1:** Basic characteristics and quality evaluation results of the included documents

Author (year)	Country	Age	Research type	Number of stroke types		Aphemia judgment method	Document quality score
				ischemia	bleed		
Bhatnagar(2002) [14]	India	-	cohort	-	-	①	6
Carlo(2002) [15]	Italy	71.8 ± 12.6	cohort	2740	545	-	7
Godefroy(2002) [16]	France	62 ± 16	cohort	263	45	②	7
Trapl(2004) [17]	Austria	71.6 ± 12.08	cohort	-	-	③	6
Pedersen(2004) [18]	Denmark	75.8 ± 10.4	cohort	205	-	④	8
Engelter(2006) [19]	Switzerland	-	case–control	-	-	⑤	6
Inatomi(2008) [8]	Japan	-	cohort	130	0	⑥	7
Kyrozis(2009) [20]	Greece	-	cohort	-	-	-	7
Brkić(2009) [21]	Croatia		cohort	156	38	-	6
Bersano(2009) [22]	Italy	-	cohort	2154	320	-	8
Naess(2009) [23]	Norway	-	cohort	20	0	⑦	9
Kyrozis(2009) [20]	Greece	-	cohort	158	94	-	6
Tsouli(2009) [24]	Greece	-	cohort	2022	275	-	7
Dickey(2010)[25]	Canada	73 ± 13	case–control	4237	700	⑧	6
Gialanella(2011) [26]	Italy	67.4 ± 9.8	cohort	82	23	③	7
Gialanella(2011) [27]	Italy	-	cohort	103	28	③	7
Hilari(2011) [28]	Britain	69.5 ± 12.5	cohort	75	12	⑥⑨	9
Gialanella(2011)	Italy	-	cohort	205	57	③	6
Kadojić(2012) [13]	Croatia	-	cohort	75	0	②	6
Flowers(2013) [29]	Canada	70.9 ± 13	cohort	68	0	-	7
Schnakers(2015) [30]	Belgium	66.29 ± 12.66	transverse section	4	20	③	8
Boehme(2016) [31]	America	-	cohort	934	90	⑥	7
Flowers(2017) [32]	Canada	70.8 ± 13.3	cohort	52	0	-	7
González Mc(2017) [33]	Chile	66 ± 20	cohort	142	0	-	5
Ginex(2017) [34]	Italy	75.5 ± 12.1	cohort	33	15	③	7
Lima(2019) [12]	Brazil	69.84 ± 13.88	case–control	79	0	⑥	6
Cock(2020) [7]	Belgium	74 ± 13	cohort	34	0	②	7
Gonzalez(2020) [35]	Chile	57.37 ± 15.56	cohort	-	-	②④	7
Rudolph(2020) [36]	Spain	50.05 ± 9.21	cohort	130	0	⑥	6
Xu(2021) [37]	China	61.1 ± 11.9	transverse section	180	34	④	10
Goldberg(2021) [38]	America	59.2 ± 13.0	cohort	122	0	②④	7
Grönberg(2022) [39]	Sweden	-	case–control	91	0	⑥	8
Lin(2022) [40]	Taiwan	-	cohort	11,494	5569	⑥	8
Grönberg(2022) [4]	Sweden	-	cohort	308	0	⑩	7
Wilson(2022) [41]	America	-	cohort	174	68	-	8
Brogan(2023) [42]	Australian	-	cohort	1658	206	-	7

① AIIMS aphasia examination test; ② Boston Diagnostic Aphasia Test (BDAE); ③ Aachen aphasia test (AAT); ④ Western Aphasia Test (WAB); ⑤ Differential diagnosis of Minnesota aphasia; ⑥ National Institutes of Health Stroke Scale (NIHSS); ⑦ Pet-name ruby Afasi Norwegian Standard (NGA); ⑧ Canadian Neurometric Scale; ⑨ French Aphasia Screening Test (FAST); ⑩Quick Aphasia Battery(QAB)

### Risk of bias in included studies

A total of 36 articles were included, including 27 high-quality articles and 9 medium-quality articles. The main factor affecting the quality of cohort studies and cross-sectional studies is the follow-up part, and eight studies considered the relative long-term and adequacy of follow-up. Comparability between exposed/case groups and nonexposed/control groups is also an important part of the score. Fourteen studies fully compared the general data affecting the results and considered the influence of confounding factors, which improved the referential of the study. Only 18 studies accurately provided the statistical data of PSA patients' age, and other studies failed to write according to the standard, which affected the comparability score between groups and reduced the accuracy of experimental results.

## Meta-analysis

### Sex and risk of poststroke aphasia

**Fig. 2:**
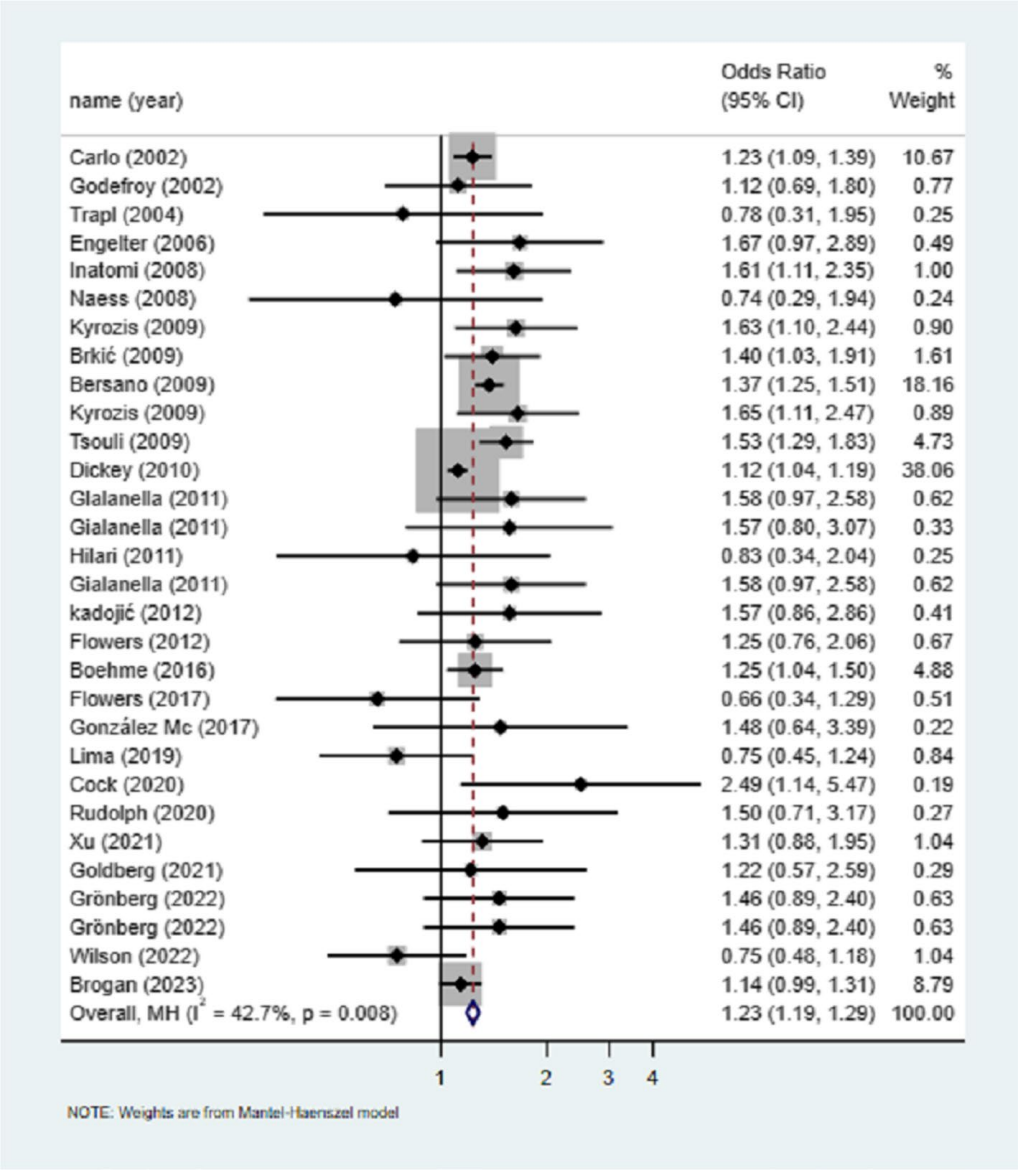
Sex and the risk of poststroke aphasia

Taking female PSA group after stroke as the exposure group, 31 studies were analyzed by meta-analysis of binary variables, and I^2^ = 84.5% (Supplementary Fig. 1) thought that the heterogeneity was high, and the source of heterogeneity was not shown by sensitivity analysis (Supplementary Fig. 2). Excluding Lin et al.' s research [40], the heterogeneity is less than 50%. The results show that the risk of PSA in women is higher than that in men, which is 1.23 times that in men (OR = 1.23, 95%CI = 1.19–1.29, *P* < 0.05). See Fig. 2.

### Sex and incidence of poststroke aphasia

**Fig. 3:**
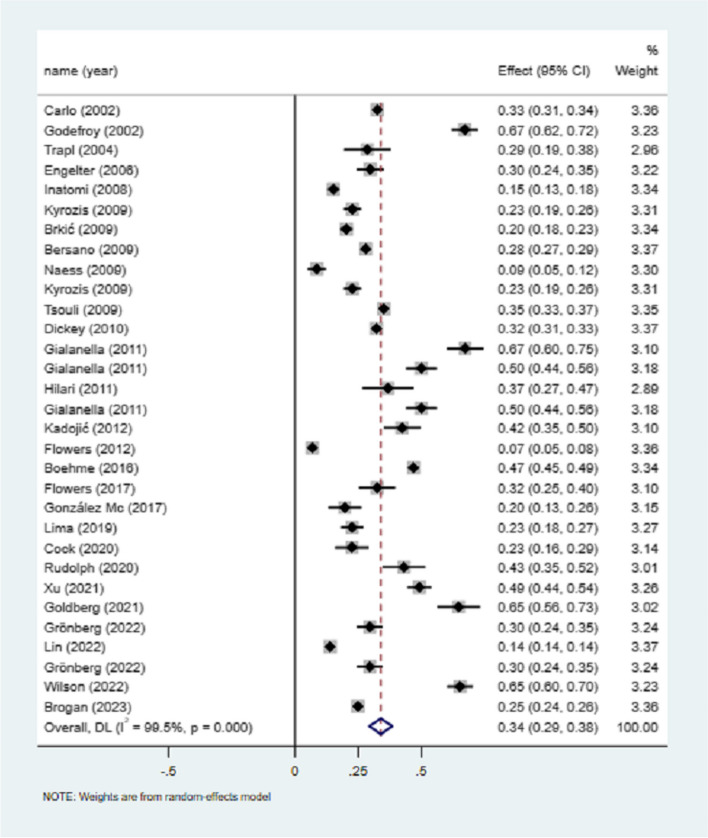
Incidence of poststroke aphasia

31 studies mentioned the total number of stroke and PSA, and the meta-analysis of utilization rate showed that the incidence of PSA was 34% (I^2^ = 99.5%, 95%CI = 0.29–0.38, *P* < 0.001), as shown in Fig. 3. Sensitivity analysis did not find the source of heterogeneity (Supplementary Fig. 3).

**Fig. 4:**
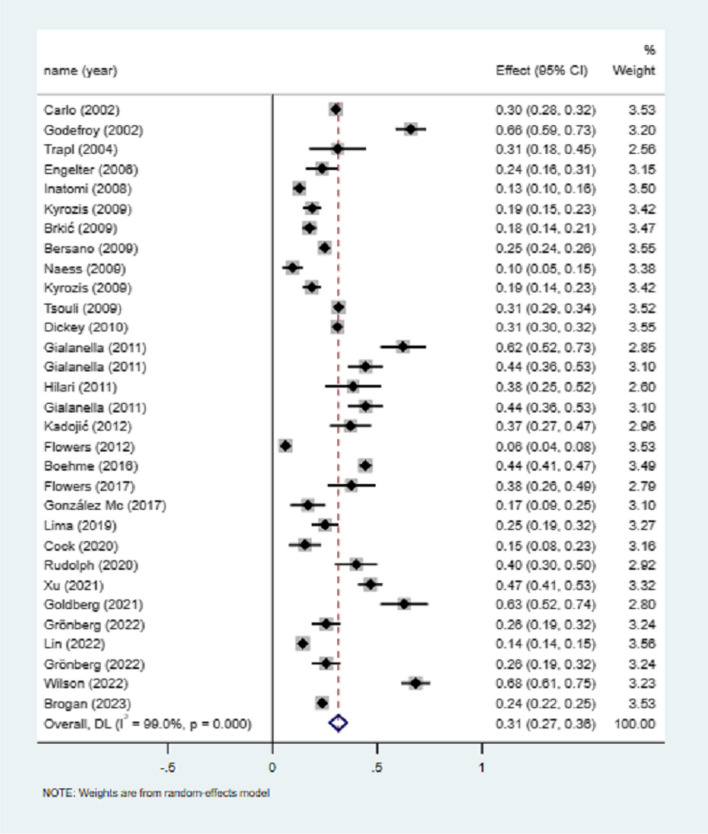
Incidence of poststroke aphasia in male

31 studies mentioned male stroke and the total number of PSA, and the meta-analysis of the utilization rate showed that the incidence of male PSA was 31% (I^2^ = 99.0%, 95%CI = 0.27–0.36, *P* < 0.001), as shown in Fig. 4. Sensitivity analysis did not find the source of heterogeneity (Supplementary Fig. 4).

**Fig. 5:**
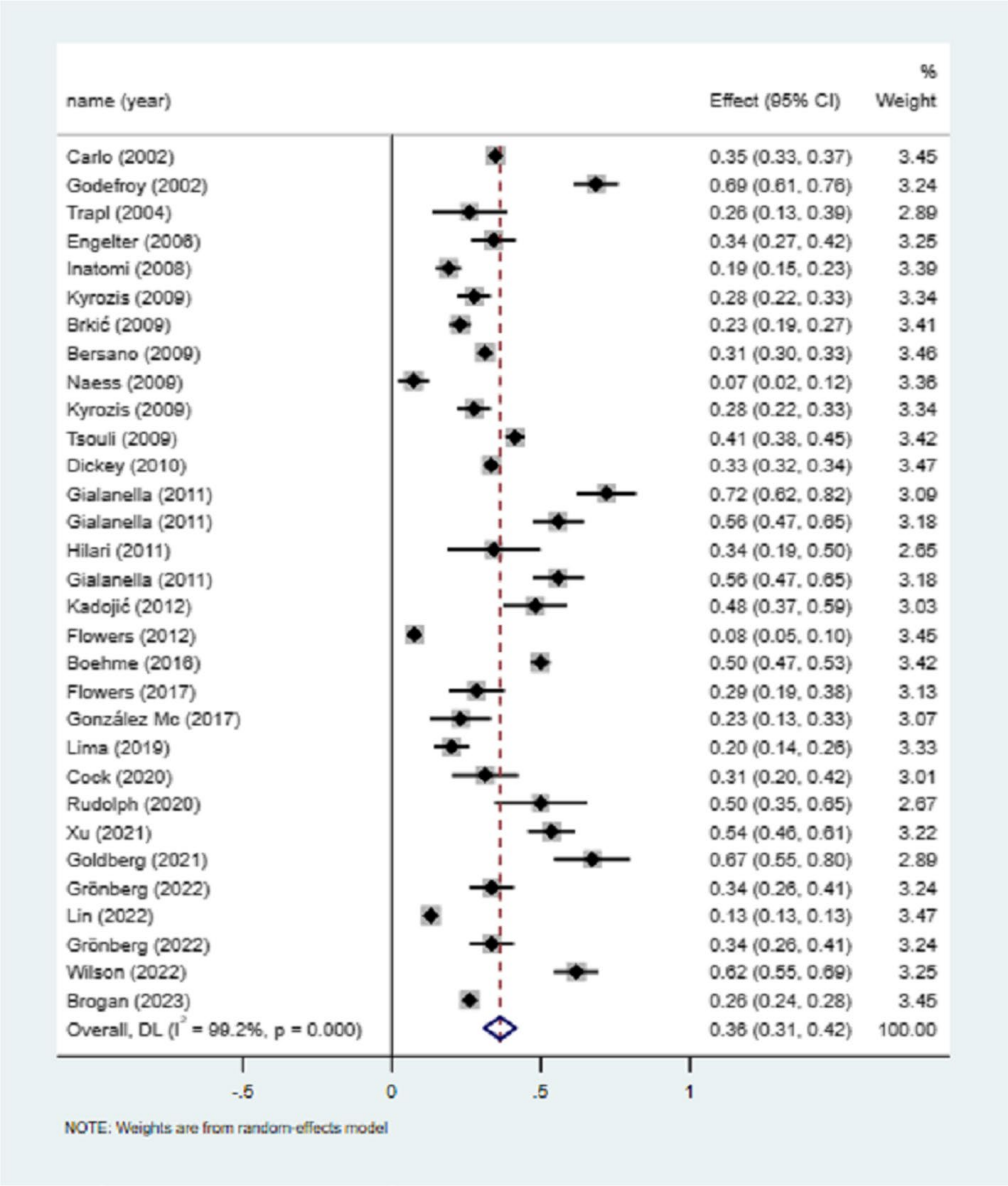
Incidence of poststroke aphasia in female

31 studies mentioned the total number of female stroke and PSA, and the meta-analysis of utilization rate showed that the incidence of female PSA was 36% (I^2^ = 99.2%, 95%CI = 0.31–0.42, *P* < 0.001), as shown in Fig. 5. Sensitivity analysis did not find the source of heterogeneity (Supplementary Fig. 5).

### Sex and risk, incidence of type of poststroke aphasia

#### Global aphasia

**Fig. 6:**
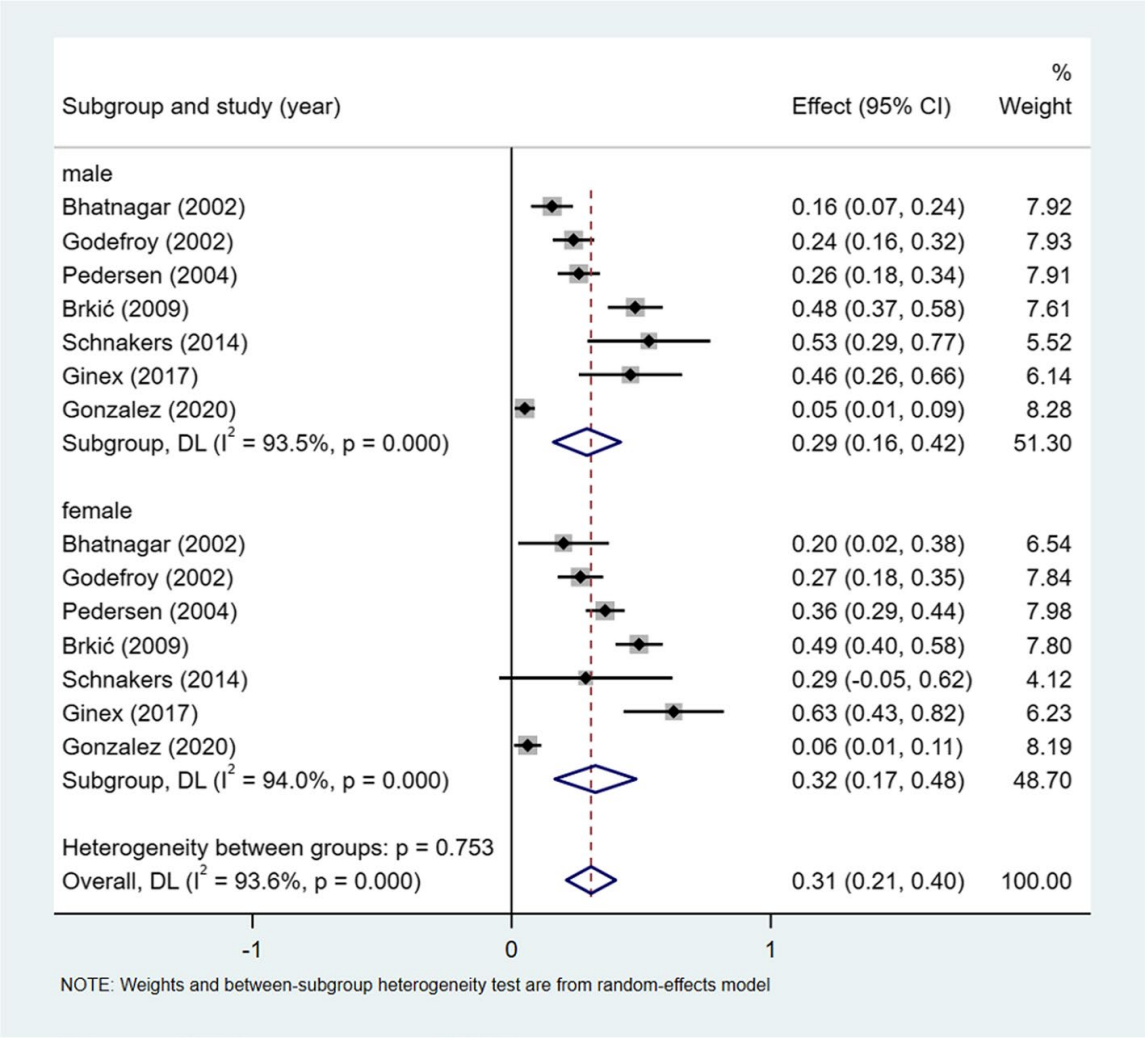
Incidence of global aphasia

A meta-analysis of dichotomous variables in 7 studies showed that the risk of global aphasia was 1.20 times higher in female than in male (OR = 1.27, 95%CI = 0.95–1.70, *P* = 0.734), see Supplementary Fig. 6. A meta-analysis of rates in 7 studies and by gender subgroup, showed the incidence of global aphasia 31% (I^2^ = 93.6%, 95%CI = 0.21–0.40, *P* < 0.001), 29% (I^2^ = 93.5%,95%CI = 0.16–0.42, *P* < 0.001) in male, and 32% (I^2^ = 94.0%,95%CI = 0.17–0.48, *P* < 0.001) in female, see Fig. 6.

### Broca aphasia

**Fig. 7:**
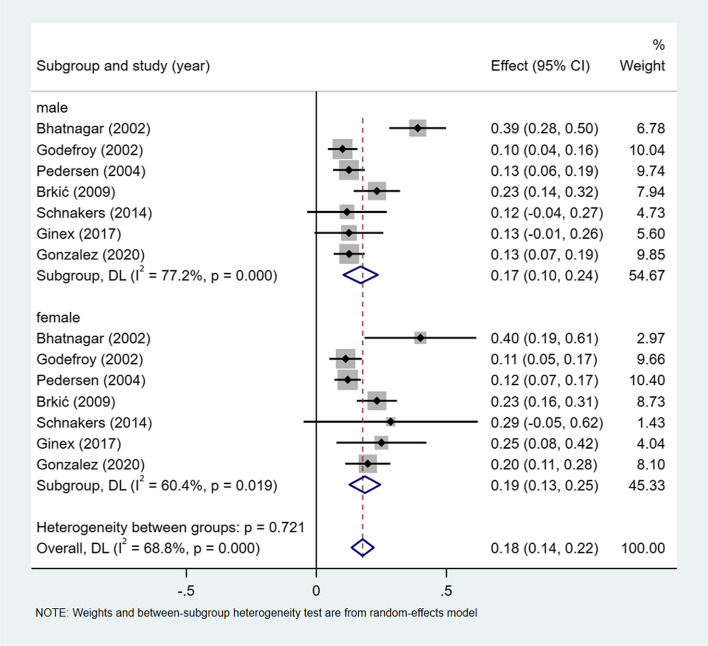
Incidence of broca aphasia

A meta-analysis of dichotomous variables in 7 studies showed that the risk of broca aphasia was 1.20 times higher in female than in male (OR = 1.20, 95%CI = 0.86–1.69, *P* = 0.816), see Supplementary Fig. 7. A meta-analysis of rates in 7 studies and by gender subgroup, showed the incidence of broca aphasia 18% (I^2^ = 68.8%, 95%CI = 0.14–0.22, *P* < 0.001), 17% (I^2^ = 77.2%,95%CI = 0.10–0.24, *P* < 0.001) in male, and 19% (I^2^ = 60.4%,95%CI = 0.13–0.25, *P* < 0.05) in female, see Fig. 7.

### Anomic aphasia

**Fig. 8:**
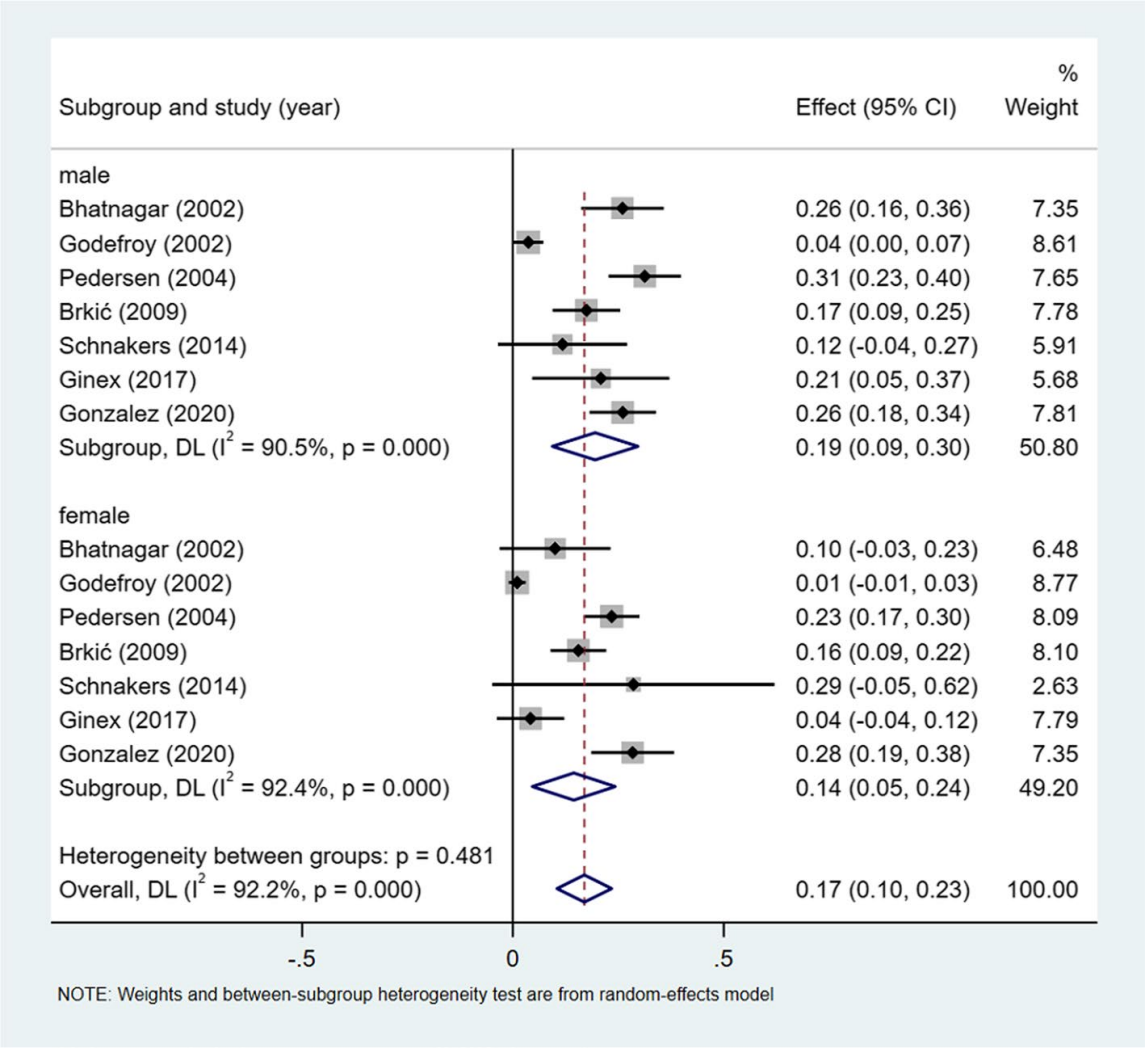
Incidence of anomic aphasia

A meta-analysis of dichotomous variables in 7 studies showed that the risk of anomic aphasia was 1.33 times higher in male than in female (OR = 1.33, 95%CI = 0.95–1.86, *P* = 0.305), see Supplementary Fig. 8. A meta-analysis of rates in 7 studies and by gender subgroup, showed the incidence of anomic aphasia 17% (I^2^ = 92.2%, 95%CI = 0.10–0.23, *P* < 0.001), 19% (I^2^ = 90.5%, 95%CI = 0.09–0.30, *P* < 0.001) in male, and 14% (I^2^ = 92.4%, 95%CI = 0.05–0.24, *P* < 0.001) in female, see Fig. 8.

### Wernicke aphasia

**Fig. 9:**
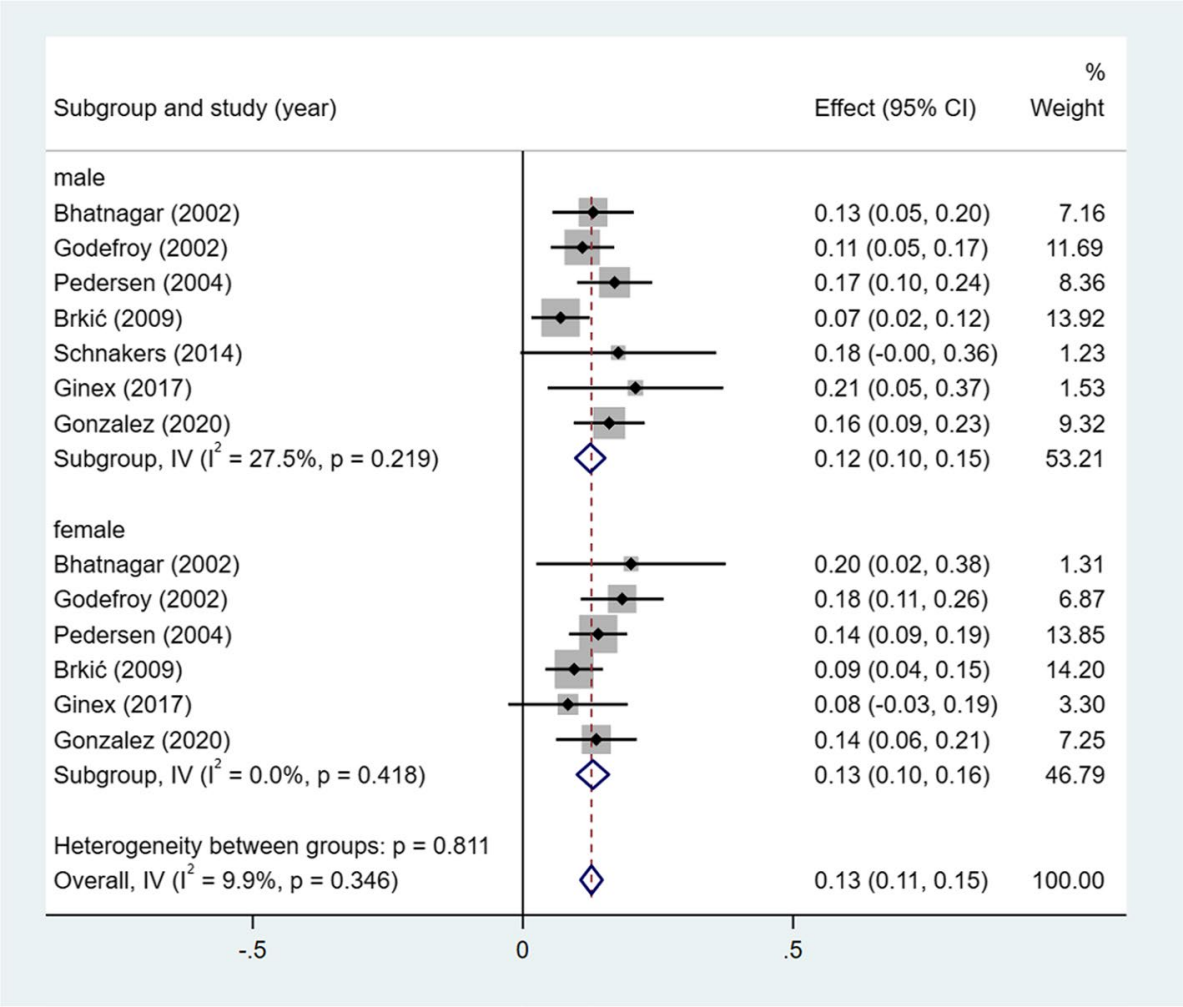
Incidence of wernicke aphasia

A meta-analysis of dichotomous variables in 7 studies showed that the risk of wernicke aphasia was 1.02 times higher in female than in male (OR = 1.02, 95%CI = 0.71–1.47, *P* = 0.427), see Supplementary Fig. 9. A meta-analysis of rates in 7 studies and by gender subgroup, showed the incidence of wernicke aphasia 13% (I^2^ = 9.9%, 95%CI = 0.11–0.15, *P* = 0.346), 13% (I^2^ = 27.5%, 95%CI = 0.09–0.16, *P* = 0.219) in male, and 13% (I^2^ = 0.0%, 95%CI = 0.10–0.16, *P* = 0.418) in female, see Fig. 9.

### Transcortical mixed aphasia

**Fig. 10:**
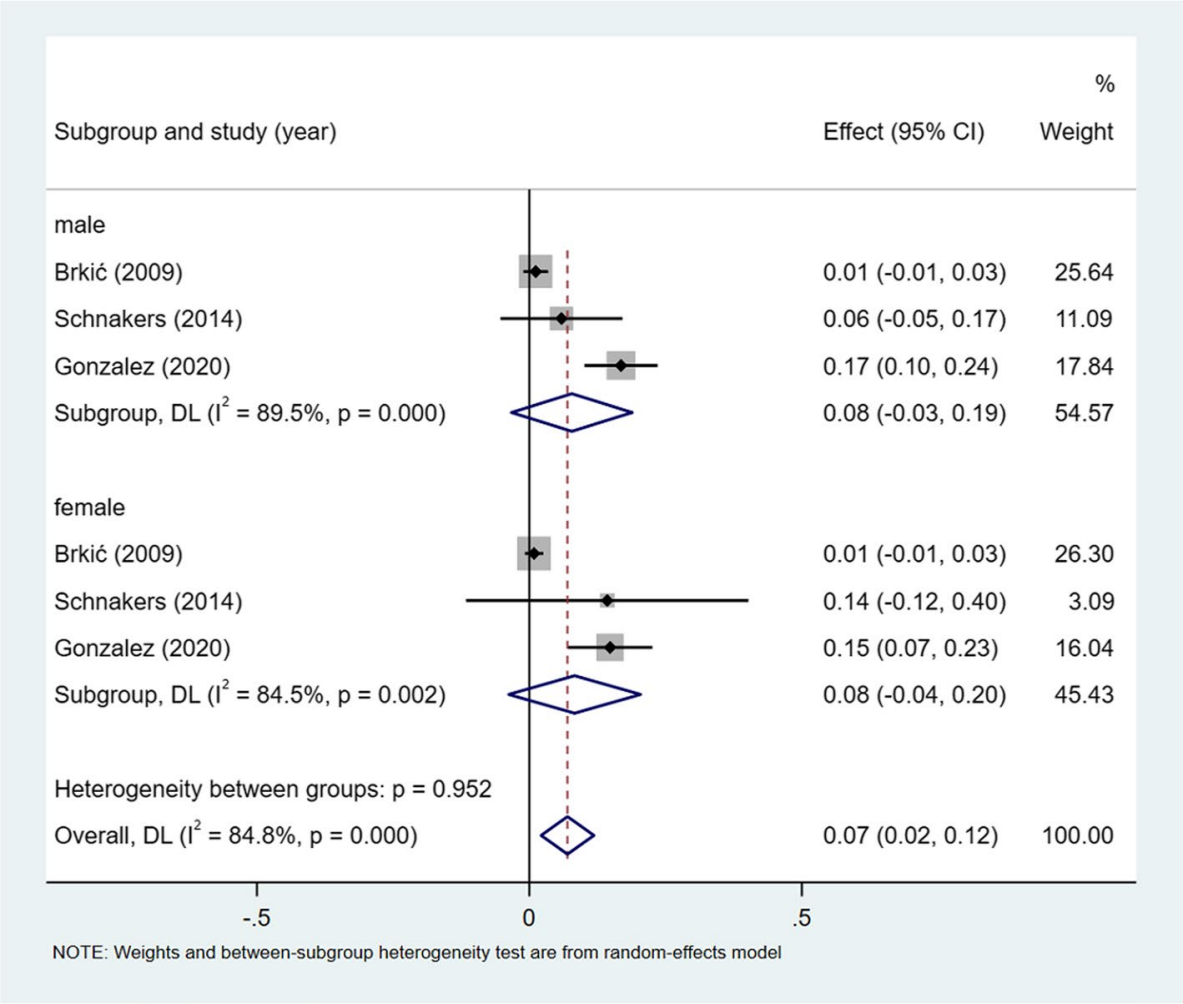
Incidence of transcortical mixed aphasia

A meta-analysis of dichotomous variables in 3 studies showed that the risk of transcortical mixed aphasia was 1.10 times higher in male than in female (OR = 1.10, 95%CI = 0.53–2.27, *P* = 0.756), see Supplementary Fig. 10. A meta-analysis of rates in 3 studies and by gender subgroup, showed the incidence of transcortical mixed aphasia 7% (I^2^ = 84.8%, 95%CI = 0.02–0.12, *P* < 0.001), 8% (I^2^ = 89.5%, 95%CI = 0.03–0.19, *P* < 0.001) in male, and 8% (I^2^ = 84.5%, 95%CI = 0.04–0.20, *P* < 0.001) in female, see Fig. 10.

### Conductive aphasia

**Fig. 11:**
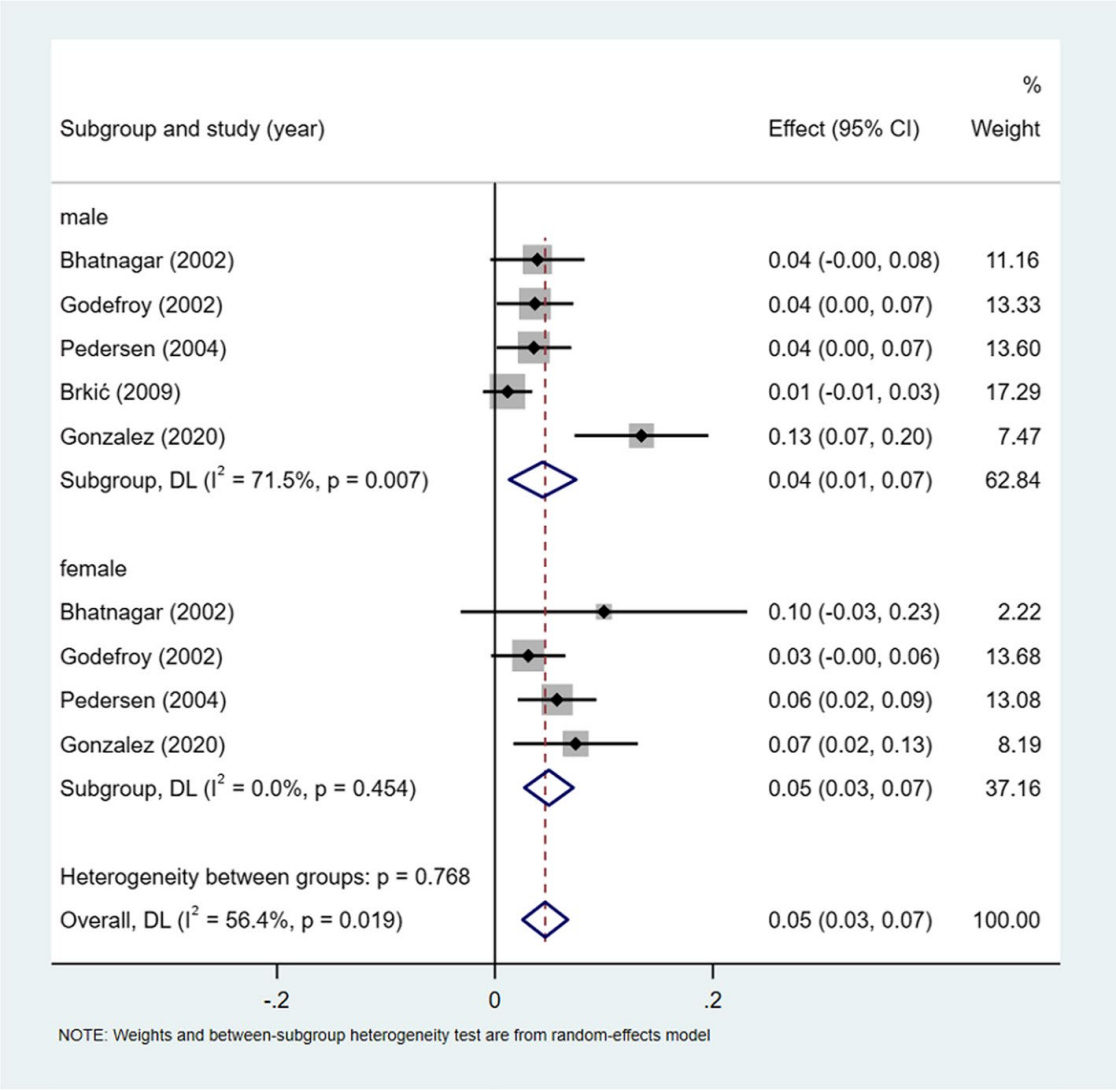
Incidence of conductive aphasia

A meta-analysis of dichotomous variables in 5 studies showed that the risk of conductive aphasia was 1.15 times higher in male than in female (OR = 1.15, 95%CI = 0.62–2.11, *P* = 0.380), see Supplementary Fig. 11. A meta-analysis of rates in 5 studies and by gender subgroup, showed the incidence of conductive aphasia 5% (I^2^ = 56.4%, 95%CI = 0.21–0.40, *P* < 0.05), 4% (I^2^ = 71.5%, 95%CI = 0.01–0.07, *P* < 0.05) in male, and 5% (I^2^ = 0.0%, 95%CI = 0.03–0.07, *P* = 0.454) in female, see Fig. 11.

### Transcortical sensory aphasia

**Fig. 12:**
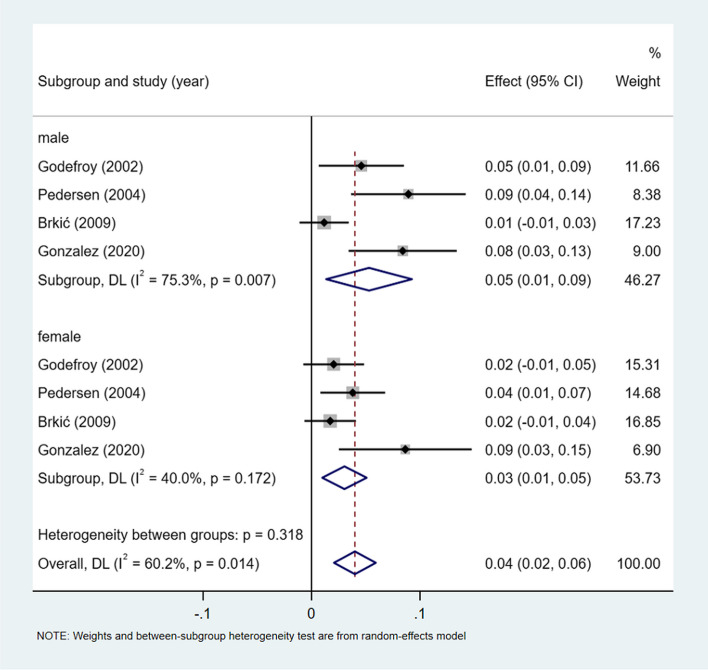
Incidence of transcortical sensory aphasia

A meta-analysis of dichotomous variables in 4 studies showed that the risk of transcortical sensory aphasia was 1.54 times higher in male than in female (OR = 1.54, 95%CI = 0.82–2.89, *P* = 0.514), see Supplementary Fig. 12. A meta-analysis of rates in 4 studies and by gender subgroup, showed the incidence of transcortical sensory aphasia 4% (I^2^ = 60.2%, 95%CI = 0.02–0.06, *P* < 0.05), 5% (I^2^ = 75.3%, 95%CI = 0.02–0.06, *P* < 0.05) in male, and 3% (I^2^ = 40.0%, 95%CI = 0.01–0.05, *P* = 0.172) in female, see Fig. 12.

### Transcortical motor aphasia

**Fig. 13:**
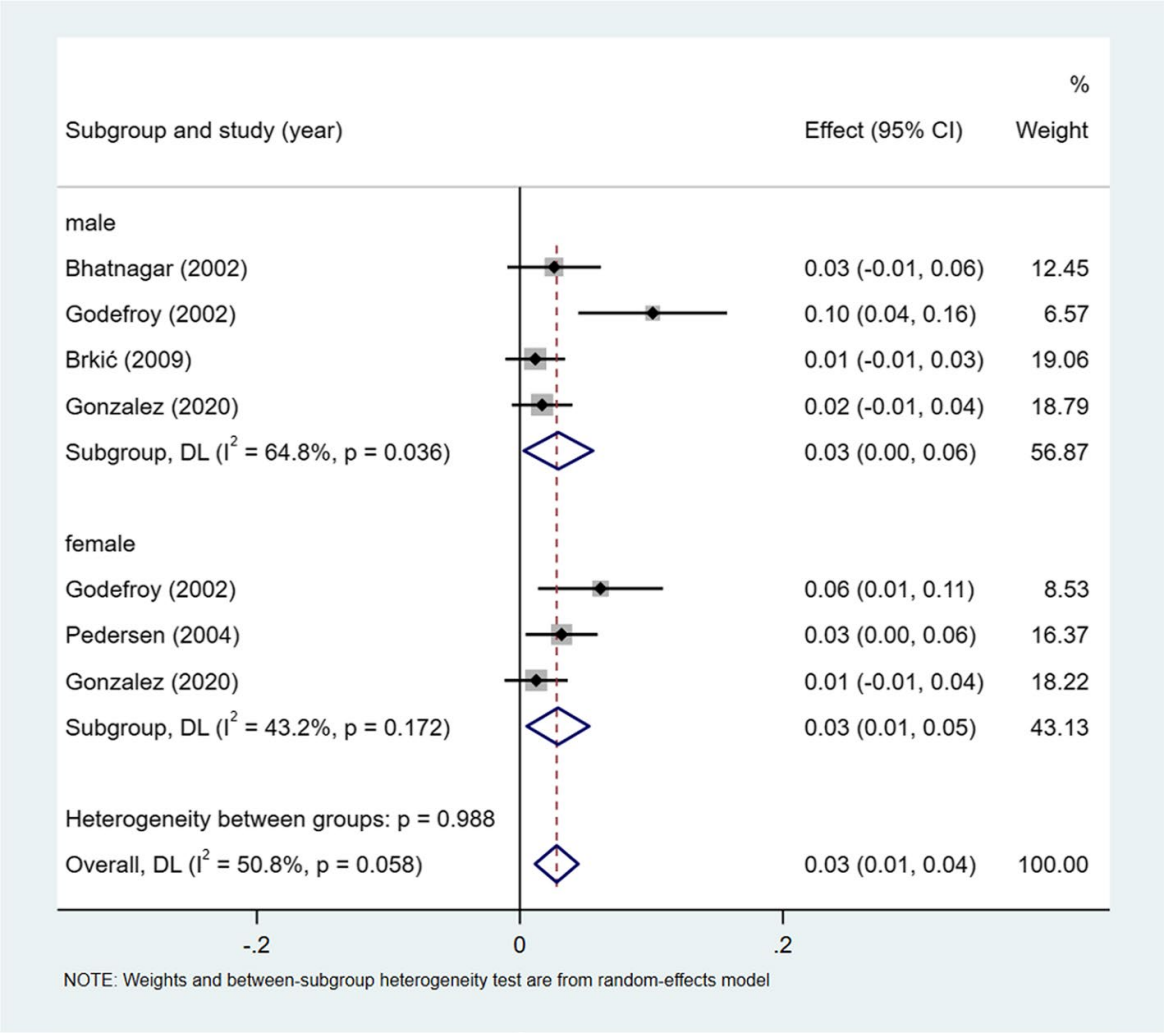
Incidence of transcortical mortor aphasia

A meta-analysis of dichotomous variables in 5 studies showed that the risk of transcortical motor aphasia was 1.21 times higher in male than in female (OR = 1.21, 95%CI = 0.55–2.70, *P* = 0.498), see Supplementary Fig. 13. A meta-analysis of rates in 5 studies and by gender subgroup, showed the incidence of transcortical motor aphasia 3% (I^2^ = 50.8%, 95%CI = 0.01–0.04, *P* = 0.058), 3% (I^2^ = 64.8%, 95%CI = 0.00–0.06, *P* < 0.05) in male, and 3% (I^2^ = 43.2%, 95%CI = 0.01–0.05, *P* = 0.172) in female, see Fig. 13.

### Other type of aphasia

**Fig. 14:**
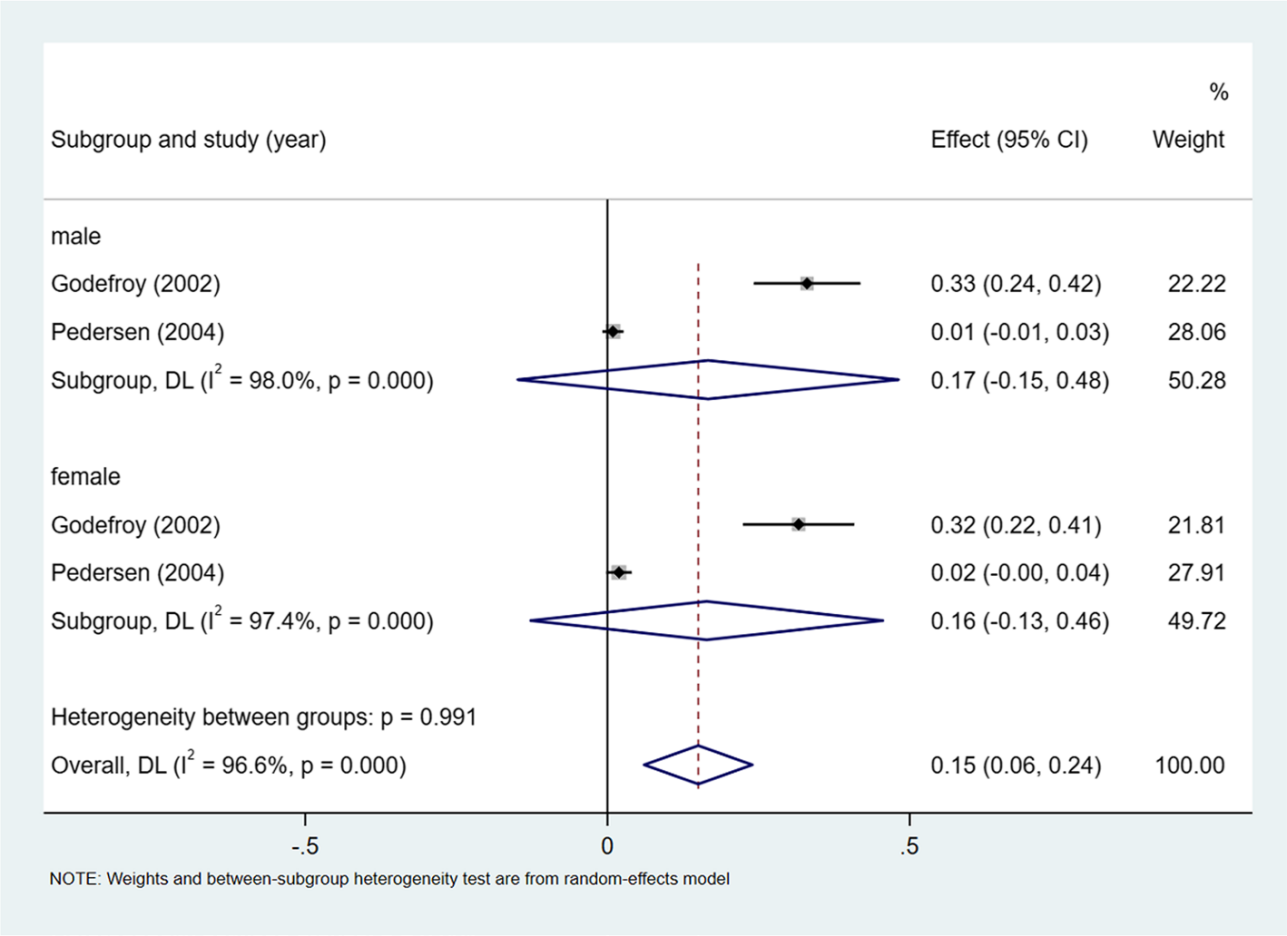
Incidence of other type of aphasia

A meta-analysis of dichotomous variables in two studies showed that sex had no predictive significance of developing risk in other types of aphasia (OR = 1.00, 95%CI = 0.57–1.74, *P* = 0.489), as shown in Supplementary Fig. 14. A meta-analysis of rates in 2 studies and by gender subgroup, showed the incidence of other types of aphasia 15% (I^2^ = 96.6%, 95%CI = 0.06–0.24, *P* < 0.001), 17% (I^2^ = 98.0%, 95%CI = 0.15–0.48, *P* < 0.001) in male, and 16% (I^2^ = 97.4%, 95%CI = 0.13–0.46, *P* < 0.001) in female, see Fig. 14.

## Discussion

In this paper, 168,259 stroke patients were analyzed by meta-analysis, and the results showed that the risk of PSA in female was significantly higher than that in male, which was 1.23 times that in male. This meta-analysis showed that the total incidence of PSA was 34%, which was within the range of 13%-35% estimated by previous studies [28, 29]. The incidence of PSA is affected by diagnostic criteria, and the results are different. At present, there is no unified standard for PSA diagnosis. The aphasia quotient is calculated by weighting the four tests of spontaneous language, auditory comprehension, retelling and naming. The lower the score, the more serious the injury. In other countries, the ninth item of the NIHSS is used to evaluate PSA, and the score is simply given according to the severity of aphasia [8]. The higher the score, the more serious the injury, and it is easy to miss the diagnosis of patients with mild aphasia [39].

The gender difference in the incidence of PSA is related to the bilateralization of language functional areas in the female brain. In the population, 96%-99% of right-handed people and 60% of left-handed people are located in the left brain [43]. In recent years, more scholars have tried to explore the importance of the activation of the right brain region in improving aphasia. Chang et al.[44] found that a patient with global aphasia without hemiplegia after left cerebral stroke can restore oral fluency by activating the right frontal lobe. In the critical period when fetal language controls the development of brain regions, it is regulated by sex hormones, resulting in differences in hemispheric structure and language processing [45]. The development of the brain tends to mature in adolescence, when the testosterone level of boys is 20 times that of girls, the estradiol level has little difference. Abnormal sex hormones are often accompanied by speech disorders [46, 47]. Functional magnetic resonance imaging (fMRI) was used to study the activated brain regions in language processing, and it was found that men only activated the left side. While women activated both sides, suggesting that women activated extensive brain regions, resulting in gender differences in PSA incidence [48].

There are also studies that the difference in incidence between men and women is related to the age of onset, and there is an enormous age difference between men and women in PSA.[20] This showed that women are often older than men when they are sick. The analysis by Wallentin et al.[11] shows that the sex ratio of PSA in different age groups is significantly different. The sex ratio of patients under 64 years old was 1.04, that of patients aged 64–74 years old was 1.08, that of patients aged 75–84 years old was 1.16 and that of patients over 84 years old was 1.22. With increasing age, the prevalence of PSA in women gradually increased. Women have more ability to keep healthy than men, the age-standardized mortality rate of women is lower than that of men in the same year.There were more women in the long-lived population [49]. Age is a risk factor for PSA, and 75%-80% of strokes occur in patients over 65 years old [20]. The median age of female(78) onset is higher than that of male(71) onset.[50] Kadojić et al.[13] found that the prevalence of PSA in women over 85 years old increased by 7.5 times compared with that in men over 65 years old and only increased by 2 times.

Although it is generally believed that the location and size of stroke are the decisive factors of PSA types. Some studies have shown that 63.5% of PSA classification is not completely related to the damage of classical language functional areas, and it needs to be supported by a complete brain semantic network system [51–53]. PSA often causes other serious poststroke complications, among which patients with nonfluent aphasia are usually accompanied by poor motor function and prognosis recovery [26, 54, 55]. There was no authoritative classification standard for PSA types, and 26.5% of aphasia cases cannot be classified [52].

The NIHSS score of women at admission is generally lower than that of men, and the degree of stroke is more serious, which gradually increases with age [50]. Lee and others[56] think that NIHSS score can predict aphasia quotient, indicating that stroke severity is an important variable of PSA degree. Global aphasia is the most common PSA type [4, 13]. Due to the connectivity of the whole brain language network and the abnormal connection mode of key language intervals, its language fluency, listening comprehension and retelling ability are poor, which is the most serious aphasia [35]. The language fluency of different sexes is affected by the volume and density of gray matter [57]. The brain volume, volume and density of gray matter in men are larger than those in women, and women are more likely to damage more gray matter in severe stroke, which affects language fluency [58]. During stroke, diffuse depolarization and cytotoxic edema occur in the gray matter of the brain, which mediates the death of neurons and damages the gray matter [59]. This paper thought that the incidence of female (28%) global aphasia was significantly higher than that of male (27%), but there was no research on the influence and correlation of sex in a large sample of patients.

Ellis et al.[60] showed that age is related to PSA types, and the incidence of broca aphasia is higher in young people. The increase in age is accompanied by extensive changes in brain structure, which often leads to a decline in language competence, but the understanding function is preserved [61]. The most classic type of nonfluent aphasia is broca aphasia, which is characterized by poor oral fluency and retelling ability and relatively good listening and understanding ability, which damages broca's brain area and affects the continuity of language [62, 63]. After analysis by Sharma et al.[10], it was found that the incidence of broca aphasia in men (27.4%) was significantly higher than that in women (19.2%), with an average age of 61.9 years. At present, the mechanism is not clear. Sharma[10] thinks that in addition to the differences in onset age and hemispheric structure between men and women, the damaged vascular area also needs to be further explored. Some studies have also shown that the incidence of broca aphasia in men in all age groups is higher than that in women, and further research and discussion are needed.

Anomic aphasia is aphasia characterized by an inability to name, and other language abilities are relatively complete. The incidence rate is relatively low in acute stroke, and it is usually the outcome type of other aphasia. There was a significant positive correlation between naming accuracy and language span length (r = 0.732), and word naming is considered a short-term memory of speech, which is influenced by the temporary activation of language representation [64]. Female scores in short-term memory are significantly higher than male, their word recall ability and the number of single memories are superior, and their recovery after injury is also better than that of male. In this paper, the incidence of anomic aphasia in men (18%) was higher than that in women (16%), which is closely related to the difference in naming between men and women before injury [65, 66].

The incidence of transcortical motor aphasia, transcortical mixed aphasia, other type of aphasia, conductive aphasia and transcortical sensory aphasia is less than 10%. However, due to the relatively low incidence, previous observational studies have not reached the same conclusion, and the specific mechanism needs to be explored by more scholars. Other types of aphasia in this paper include cross-aphasia, isolated aphasia and basal ganglia aphasia. But the conclusion after meta-analysis suggests that there is no difference between the sexes, and there is no statistical significance. Perhaps because the number of people suffering from other aphasia is relatively small, it is not considered that the incidence of other aphasia is affected by gender. There is no significant gender difference among wernicke aphasia, conductive aphasia and transcortical sensory aphasia, and it is not considered that gender is related to them.

Although sex can't predict the occurrence of stroke, the incidence of stroke in male is higher than that in female worldwide [67]. This leads researchers to pay more attention to reducing the risk factors of stroke in male, while ignoring the physical condition of female. Through meta-analysis, this study holds that the probability of aphasia in female after stroke is higher than that in male, and the severity is also higher than that in male, which is caused by the damage of language function areas in both brains of women after stroke. This requires medical workers to pay special attention to training female right language function area in daily propaganda to prevent stroke and serious PSA. The gender difference in the types of PSA can guide doctors to use physical therapy equipment to carry out early rehabilitation of PSA in the early stage of stroke when patients are in a state of continuous coma. Of course, this requires the auxiliary examination of imaging to confirm the damaged brain area of the patient.

### Study limitations

In this paper, a large number of similar studies are analysed, but due to the limitations of literature types. It is impossible to correct for confounding factors such as age, stroke type and injured hemisphere. Subsequent scholars can eliminate the influence of confounding factors through large sample size investigations and studies and obtain more accurate results. The included studies come from 19 countries and regions, and there are great differences among them. It is difficult to completely eliminate their heterogeneity through statistical methods, and the statistical analysis from international cooperation is expected in the future.

## Conclusion

In summary, there are differences in the incidence and types of PSA between male and female.  The risk of PSA in female is higher than that in male, which is related to the activation of bilateral brain language functional areas and the age at onset. The incidence of global aphasia and broca aphasia is high in female, and the incidence of anomic aphasia is higher in male. Different sexes cause differences in aphasia types, which are influenced by the degree of gray matter injury, age of onset and activation of language representation. This study can guide clinical workers to carry out gender-specific preventive intervention for people at risk of stroke, and to recover as soon as possible in different aphasia types for different sexes after stroke.

### Supplementary Information

**Additional file 1. Supplementary Figure 1.** Sex and the risk of poststroke aphasia (before exclusion). **Supplementary Figure 2.** Sensitivity analysis of sex and the risk of poststroke aphasia. **Supplementary Figure 3.** Sensitivity analysis of incidence of poststroke aphasia. **Supplementary Figure 4.** Sensitivity analysis of incidence of poststroke aphasia in male. **Supplementary Figure 5.** Sensitivity analysis of incidence of poststroke aphasia in female. **Supplementary Figure 6.** Sex and the risk of global aphasia. **Supplementary Figure 7.** Sex and the risk of broca aphasia. Supplementary Figure 8. Sex and the risk of anomic aphasia. **Supplementary Figure 9.** Sex and the risk of wernicke aphasia. **Supplementary Figure 10.** Sex and the risk of transcortical mixed aphasia. Supplementary Figure 11. Sex and the risk of conductive aphasia. **Supplementary Figure 12.** Sex and the risk of transcortical sensory aphasia. **Supplementary Figure 13.** Sex and the risk of transcortical mortor aphasia. **Supplementary Figure 14.** Sex and the risk of other type of aphasia.

## Data Availability

The datasets generated and/or analysed during the current study are not publicly available due to space limitation but are available from the corresponding author on reasonable request.
