# Supplementary material for: Meta-analysis and systematic review of the relationship between sex and the risk or incidence of poststroke aphasia and its types
Source: BMC Geriatr. 2024 Mar 4;24:220. doi: 10.1186/s12877-024-04765-0 (PMC10910787; doi:10.1186/s12877-024-04765-0)
Supplement: Supplementary file 1 — Additional file 1. Supplementary Figure 1. Sex and the risk of poststroke aphasia (before exclusion). Supplementary Figure 2. Sensitivity analysis of sex and the risk of poststroke aphasia. Supplementary Figure 3. Sensitivity analysis of incidence of poststroke aphasia. Supplementary Figure 4. Sensitivity analysis of incidence of poststroke aphasia in male. Supplementary Figure 5. Sensitivity analysis of incidence of poststroke aphasia in female. Supplementary Figure 6. Sex and the risk of global aphasia. Supplementary Figure 7. Sex and the risk of broca aphasia. Supplementary Figure 8. Sex and the risk of anomic aphasia. Supplementary Figure 9. Sex and the risk of wernicke aphasia. Supplementary Figure 10. Sex and the risk of transcortical mixed aphasia. Supplementary Figure 11. Sex and the risk of conductive aphasia. Supplementary Figure 12. Sex and the risk of transcortical sensory aphasia. Supplementary Figure 13. Sex and the risk of transcortical mortor aphasia. Supplementary Figure 14. Sex and the risk of other type of aphasia. [file 12877_2024_4765_MOESM1_ESM.zip › supplementary_figure/Supplementary Figure 2. Sensitivity analysis of sex and the risk of poststroke aphasia.doc]

**Supplementary Figure 2. Sensitivity analysis of sex and the risk of poststroke aphasia**
